# The associations of poor psychiatric well-being among incarcerated men with injecting drug use histories in Victoria, Australia

**DOI:** 10.1186/s40352-018-0059-4

**Published:** 2018-01-13

**Authors:** Reece Cossar, Mark Stoové, Stuart A. Kinner, Paul Dietze, Campbell Aitken, Michael Curtis, Amy Kirwan, James R. P. Ogloff

**Affiliations:** 1 Centre for Forensic Behavioural Science, Swinburne University of Technology & Forensicare, Melbourne, Australia; 2 Behaviours and Health Risks, Burnet Institute, Melbourne, Australia; 3 School of Public Health and Preventive Medicine, Monash University, Melbourne, Australia; 4 Centre for Adolescent Health, Murdoch Children’s Research Institute, Melbourne, Australia; 5 Centre for Mental Health, Melbourne School of Population and Global Health, University of Melbourne, Melbourne, Australia; 6 Griffith Criminology Institute, Griffith University, Brisbane, Australia; 7 Mater Research Institute-UQ, University of Queensland, Mount Gravatt, Australia

**Keywords:** Injecting drug use, Dual diagnosis, GHQ-12, Prisoner health

## Abstract

**Background:**

Dual substance dependence and psychiatric and psychological morbidities are overrepresented in prison populations and associated with reoffending. In the context of an increasing prison population in Australia, investigating the needs of vulnerable people in prison with a dual diagnosis can help inform in-prison screening and treatment and improve prison and community service integration and continuation of care. In this study we quantified psychiatric well-being in a sample of people in prison with a history of injecting drug use in Victoria, Australia, and identified factors associated with this outcome.

**Methods and Results:**

Data for this paper come from baseline interviews undertaken in the weeks prior to release as part of a prospective cohort study of incarcerated men who reported regular injecting drug use prior to their current sentence. Eligible participants completed a researcher-administered structured questionnaire that canvassed a range of issues. Psychiatric well-being was assessed using the 12-item General Health Questionnaire (GHQ-12) and potential correlates were included based on a review of the literature. Of the 317 men included for analyses, 139 were classified as experiencing current poor psychiatric well-being. In the multivariate model using modified logistic regression, history of suicide attempt (aOR = 1.36, 95%CI 1.03–1.78), two or more medical conditions (aOR = 1.87, 95%CI 1.30–2.67) and use of crystal methamphetamine in the week prior to their current sentence (aOR = 1.52, 95%CI 1.05–2.22) were statistically significantly associated with current poor psychiatric well-being.

**Conclusions:**

Comprehensively addressing the health-related needs for this vulnerable population will require a multidisciplinary approach and enhancing opportunities to screen and triage people in prison for mental health and other potential co-occurring health issues will provide opportunities to better address individual health needs and reoffending risk.

## Background

Over the past decade, the Victorian incarceration rate has increased from 100 per 100,000 adults in 2006 to 138 per 100,000 adults in 2016 (Australian Bureau of Statistics, 2016). People with a history of injecting drug use are over-represented in the Australian prison population. An estimated half of Australian prisons report a lifetime history of injecting drug use and approximately one in four reports at least one injection episode in the month prior to incarceration (Australian Institute of Health and Welfare, 2015; Reekie et al., 2014). People who inject drugs (PWID) face a heightened risk of blood-borne viral infections (Snow et al., 2014; Vescio et al., 2008), suicide (Artenie et al., 2015), overdose following release (Winter et al., 2015), and reduced personal well-being (Scott et al., 2017).

People experience disproportionate levels of psychiatric and psychological problems in prison relative to people in the community (Fazel & Baillargeon, 2010; Markowitz, 2011; Sirdifield et al., 2009). Australian research findings suggest that 44% of prison entrants have a mental health condition (Australian Institute of Health and Welfare, 2015; Schilders & Ogloff, 2016). Many present with multiple diagnoses (Sirdifield et al., 2009), including schizophrenia (Chang et al., 2015; Davis et al., 2007; Munetz et al., 2001), major mood disorders (Brink et al., 2001), anxiety disorders (Butler et al., 2011), post-traumatic stress disorder (Ogloff et al., 2017), anti-social personality disorder (Fazel & Danesh, 2002), traumatic brain injury (Durand et al., 2017; O’Rourke et al., 2016), and cognitive impairment (Dias et al., 2013; McCausland et al., 2013). Limited access to prison mental healthcare services in some correctional settings mean the needs of many people detained in prison with psychiatric or psychological problems may not be adequately met (Senior et al., 2013). Upon reception into a Victorian correctional setting all prison entrants undertake a mental health screen, typically conducted by correctional staff (Every-Palmer et al., 2014) who may be inadequately trained. In addition, specialist mental health staff may provide voluntary assessment and treatment for people detained in prison throughout Victorian correctional settings. People detained in prison requiring court ordered or involuntary assessment and treatment are transferred to the Victorian secure forensic hospital under the Mental Health Act 2014 (Vic).

Dual diagnosis – a term used to describe the co-occurrence of substance dependence with one or more psychiatric disorders – compounds the independent impact of drug dependence and mental health problems and is widely documented in prison studies (Drake & Wallach, 2000; Ogloff et al., 2004; Ogloff et al., 2015). The health profile of people in prison with dual diagnosis demands a multidisciplinary medical, psychological, and social needs approach to address extensive criminal histories (Kinner, 2006; Ogloff et al., 2015), poor quality of life (Warden et al., 2016), reduced treatment engagement (Horsfall et al., 2009), and high reoffending risk (Ogloff et al., 2004; Youssef et al., 2016). Although there are numerous studies of people in prison with injecting drug histories, comparatively few investigate co-occurring psychiatric well-being and substance use in detail. In the context of a rapidly increasing global prison population, characterising people in prison with histories of injecting drug usewith current poor psychiatric well-being could help identify unmet needs for targeted screening, improved integration of prison and community service models of care, and post-release continuation of care.

In this study we identify associations of psychiatric well-being in a cohort of incarcerated men with histories of injecting drug use, four to 6 weeks prior to release from prison. Whereas previous studies have typically examined substance use problems in people in prison with a mental health condition, our focus is on the mental health of people who inject drugs. People in prison require varying degrees of support to facilitate a successful release, and inadequate pre-release planning has been shown to be a barrier to successful reintegration from prison to community (Butzin et al., 2005). Identifying high-risk, vulnerable subpopulations oof people detained in prison prior to release could inform evidence-based policy to target transitional support services for those most at-risk of reoffending and substance use relapse.

## Method

### Study design, sample and setting

Data for this paper come from baseline interviews (*N* = 400) from the Prison and Transition Health study, which is a prospective cohort study of incarcerated men who reported regular injecting drug use prior to their current sentence. Interviews with eligible participants occur at three, 12, and 24 month time points post release from prison. Participants were recruited from one maximum, one medium, and one minimum-security correctional facility in the Australian state of Victoria to ensure a representative sample of people with regular histories of injecting drug use. Interviews occurred between the September 17, 2014 and May 24, 2016. Eligible participants (≥18 years of age, injecting at least monthly in the 6 months prior to their current sentence, expected release in the succeeding 4 weeks, and able to provide informed, written consent) completed a researcher-administered structured questionnaire that canvassed a range of issues (detailed below), typically taking 45–60 min. People on remand (pre-trial detention) were excluded from the study.

The study was approved by the Alfred Hospital Human Research Ethics Committee (79/12) and the Victorian Department of Justice Human Research Ethics Committee (CF/14/10169).

### Measures

#### Outcome measure

Psychiatric well-being was assessed using the 12-itemGeneral Health Questionnaire (GHQ-12), a well-validated screening instrument for identifying current poor psychiatric well-being. Participants rate themselves according to the degree to which they have experienced each of 12 symptoms over the past few weeks, using a four-point Likert scale. The standard method of scoring is that symptomatic responses are scored ‘1’ and non-symptomatic responses are scored ‘0’ (Goldberg, 1992; Goldberg et al., 1997), resulting in overall scores ranging from zero to 12. Mean derived cut-off thresholds are considered the most appropriate method of identifying current poor psychiatric well-being (Goldberg et al., 1998).

#### Considered correlates

Based on a review of literature, a range of potential correlates of psychiatric well-being was selected from the sociodemographic, health, criminological, alcohol and other drug use, and adverse childhood experiences domains.

##### Sociodemographic

Age (≤ 30/31–40/≥ 41), number of years of education completed (≤ 9/≥ 10 years completed), accommodation status (stable (owner occupied, private rental, or public housing)/unstable (parent/sibling/other family’s home, boarding house, crisis accommodation, staying with friend, squat, homeless), and Aboriginal and/or Torres Strait Islander identification (no/yes).

##### Health

Attempted suicide ever (no/yes), number of self-reported medical (respiratory, circulatory, musculoskeletal, neurological, hearing, vision or metabolic) conditions (none/one/≥ two), self-reported hepatitis C status (negative or don’t know/positive), and self-reported acquired brain injury (no/yes).

##### Criminological

Prison security level (maximum/medium/minimum), youth detention ever (no/yes), number of previous adult incarcerations (0–3/≥4), and Level of Service Inventory – Revised: Screening Version (Andrews & Bonta, 1998 - continuous); an eight-item quantitative screening tool used to determine level of service and supervision required to help focus treatment plans and predict future reoffending risk (Ferguson et al., 2009). A score of three or more suggests further follow-up with the individual is required.

##### Alcohol and other drug use

Age first injected (≤16/17–20/≥21), poly-drug injecting drug use in the month before their current sentence (no/yes), illicit substances used by any route of administration (heroin only/crystal methamphetamines only/heroin and crystal methamphetamines) in the week before their current sentence, and high risk alcohol consumption (at least two times per week *and* at least five or more drinks per usual drinking episode) in the year before their current sentence (no/yes).

##### Adverse childhood experiences

Removed from family home as a child ever (no/yes) and expelled from school ever (no/yes).

### Statistical analysis

Descriptive statistics were generated for each variable with respect to psychiatric well-being according to the GHQ-12. Potential correlates were examined using modified Poisson regression with robust standard errors, using odds ratios (OR/adjusted OR (aOR)) and 95% confidence intervals (95%CI). Bivariate analyses were conducted to examine associations between each potential correlate and psychiatric well-being. A multivariable model was constructed in which all potential correlates were included to determine the individual effects of each potential correlate after adjustment for others, with a complete case approach used. Statistical significance set at *p* < 0.05. All analyses were conducted with Stata 14 for Windows (StataCorp, 2015).

## Results

### Sample characteristics

Characteristics of the sample according to level of psychiatric well-being are shown in Table 1. Eighty-three participants were excluded due to incomplete data. In our sample a total GHQ-12 score of three or more (*M*_GHQ-12_ = 3.13, *SD* = 3.24) was indicative of current poor psychiatric well-being. Of the 317 men (*M*_*age*_ = 36.1, *SD =* 8.47) included for full case analysis, 139 (44%) were classified as experiencing current poor psychiatric well-being. The sample were mostly born in Australia (89%), lived in a metropolitan area before their current sentence (56%), identified as heterosexual (98%), and expected straight (under no supervision) release (70%). Methamphetamine (53%) was the drug type first injected by most, followed by heroin (37%). Nine (3%) of the men were homeless or had no fixed address prior to their current sentence. There was a high prevalence of self-reported depression (64%), anxiety (52%), schizophrenia (15%), anti-social personality disorder (9%), and bi-polar disorder (16%). Two thirds of the sample (64%) self-reportedhepatitis C infection and only one participant reported no prior adult incarceration.

**Table 1:** Participant characteristics and modified poisson regression associations with poor psychiatric well-being among incarcerated men who reported regular injecting drug use prior to their current sentence (*n* = 317)

	GHQ-12 (≥3) (*n* = 139)	GHQ-12 (≤2) (*n* = 178)	OR^a^ (95%CI)	aOR^b^ (95%CI)
Socio-demographic				
Age				
≤ 30	38 (27)	48 (28)	1	1
31–40	65 (47)	66 (38)	1.15(0.88–1.51)	1.33 (0.97–1.82)
≥ 41	36 (26)	59 (34)	0.88 (0.64–1.21)	1.08 (0.73–1.60)
Number of years of education completed				
≤ 9 years completed	58 (42)	78 (44)	1	1
≥ 10 years completed	81 (58)	100 (56)	1.23 (0.95–1.59)	1.18 (0.90–1.56)
Accommodation status				
Stable	72 (52)	94 (53)	1	1
Unstable	67 (48)	84 (47)	0.96 (0.76–1.21)	0.98 (0.77–1.26)
Aboriginal and/or Torres Strait Islander identification				
No	109 (78)	151 (85)	1	1
Yes	30 (22)	27 (15)	0.82 (0.63–1.07)	0.90 (0.67–1.22)
Health				
Number of medical conditions				
None	26 (19)	64 (36)	1	1
One	42 (30)	60 (34)	1.51 (1.06–2.17)**	1.33 (0.90–1.98)
≥ Two	71 (51)	54 (30)	1.93 (1.39–2.67)**	1.87 (1.30–2.67)**
Hepatitis C status				
Negative/Don’t know	55 (40)	58 (33)	1	1
Positive	84 (60)	120 (67)	0.96 (0.69–1.08)	0.92 (0.71–1.18)
Ever attempted suicide				
No	54 (39)	110 (62)	1	1
Yes	85 (61)	68 (38)	1.59 (1.27–2.00)**	1.36 (1.03–1.78)*
Acquired brain injury				
No	109 (78)	148 (83)	1	1
Yes	30 (22)	30 (17)	1.19 (0.92–1.55)	1.02 (0.76–1.37)
Criminological				
Level of Service Inventory (*M, SD)*	5.64 (1.32)	5.31 (1.46)	1.08 (1.00–1.16)	1.08 (0.96–1.21)
Prison type				
Maximum	75 (54)	77 (43)	1	1
Medium	38 (27)	53 (30)	0.86 (0.66–1.11)	0.95 (0.72–1.26)
Minimum	26 (19)	48 (27)	0.71 (0.53–.96)*	0.96 (.66–1.41)
Number of previous adult incarcerations				
0–3	44 (32)	51 (29)	1	1
≥ 4	95 (68)	127 (71)	0.89 (0.70–1.11)	0.84 (0.63–1.11)
Youth detention ever				
No	72 (52)	63 (35)	1	1
Yes	67 (48)	115 (65)	1.05 (0.84–1.31)	0.99 (0.73–1.35)
Alcohol and other drug use				
Illicit substances used^c^				
Heroin only	22 (16)	41 (23)	1	1
Methamphetamine only	46 (33)	74 (42)	1.54 (1.08–2.19)*	1.52 (1.05–2.22*)
Both heroin and methamphetamine	71 (51)	63 (35)	1.07 (0.72–1.58)	0.96 (0.63–1.48)
High risk alcohol consumption^d^				
No	98 (71)	133 (75)	1	1
Yes	41 (29)	45 (25)	0.99 (0.77–1.27)	1.00 (0.75–1.33)
Age first injected				
≤ 16	69 (50)	76 (43)	1	1
17–20	36 (26)	59 (33)	0.93 (0.72–1.21)	0.88 (0.64–1.20)
≥ 21	34 (24)	43 (24)	0.94 (0.71–1.25)	1.00 (0.74–1.34)
Poly-drug injecting drug use^e^				
No	47 (34)	63 (35)	1	1
Yes	92 (66)	115 (65)	1.12 (0.87–1.44)	1.09 (0.81–1.47)
Adverse childhood experiences				
Removed from home as a child ever				
No	94 (68)	137 (77)	1	1
Yes	45 (32)	41 (23)	1.28 (1.01–1.61)*	1.13 (0.83–1.54)
Expelled from school ever				
No	40 (29)	64 (36)	1	1
Yes	99 (71)	114 (64)	1.21 (0.94–1.57)	1.05 (0.76–1.46)

*Note: P* values: * = *p* < .05; ** = *p* < .001; ^a^ OR = Odds ratio; ^b^ aOR = Adjusted odds ratio; ^c^At least one use, by any route of administration, in the week before their current sentence; ^d^The 12 months before their current sentence; ^e^ The week before their current sentence

### Associations with current poor psychiatric well-being

In bivariate analyses, history of suicide attempt, number of self-reported medical conditions, use of crystal methamphetamine (by any route of administration) in the week prior to their current sentence, and being removed from the home as a child were statistically significantly associated with current poor psychiatric well-being. Compared to participants released from a maximum security prison, participants recruited from the minimum security prison were less likely to have current poor psychiatric well-being.

In the multivariate model, history of suicide attempt (aOR = 1.36, 95%CI 1.03–1.78), reporting two or more medical conditions (aOR = 1.87, 95%CI 1.30–2.67) and use of crystal methamphetamine in the week prior to their current sentence (aOR = 1.52, 95%CI 1.05–2.22) remained statistically significantly associated with current poor psychiatric well-being (Table 1). Post-model testing was conducted to determine overall fit. All variables in the multivariable model showed VIF < 2, suggesting no collinearity.

## Discussion

In a sample of incarcerated men who reported regular injecting drug use prior to their current sentence, 44% were classified as experiencing current current poor psychiatric well-being as measured by the GHQ-12, indicating potential unmet need during periods of incarceration for people detained in prison regarding mental health service delivery. We found that history of suicide attempt, having two or more self-reported medical conditions, and use of crystal methamphetamine in the week prior to their current sentence were independently associated with an increased likelihood of current poor psychiatric well-being.

Findings from several studies indiate that suicide rates among people in prison and those released from prison are higher than among the general community (Artenie et al., 2015; Haglund et al., 2014; Meltzer et al., 2003; Spittal et al., 2014). In addition to standard history and examination by medical professionals, our findings support the inclusion of screening for past suicidial behaviours during reception in-take procedures. Referrals of individuals meeting criteria of past suicidal behaviours with histories of injecting drug use during this time could provide opportunities for additional assessment by advanced-trainedmental health professionals with specialist clinical expertise (Brunette et al., 2008), particularly pertaining to dual diagnosis. In correctional facilities in England and Wales analogous systems have been implemented (Hopkin et al., 2017). Local adaptions to screening processes in-prison have shown to statistically significantly improve measures of depression, anxiety and psychological distress for people detained in prison at high-risk of psychosis (Evans et al., 2017) and be relatively inexpensive (Brown, Cullen, Kooyman, & Forrester, 2015).

Two or more physical health conditions remained associated with current poor psychiatric well-being. Chronic pain and disease are well known to impact on psychiatric well-being (Burke et al., 2015), particularly for those with severe mental illness (Miller et al., 2006). Our findings highlight the need for coordination of treatment services in correctional and community settings, where the prevalence and co-occurrence of both conditions is high. The integration of physical health assessment, physical activity programs (Richardson et al., 2005), and targeted healthy well-being education (Smith et al., 2007) into community based psychiatric care services have shown to be successful models for increasing coordination of service delivery by health professionals and reducing the health burden for individuals. The introduction of similar health service delivery models in prison for people with dual diagnosis may foster the transition to community and encourage the continuation of care once released. In Victorian correctional settings, treatment programs for mental health conditions and substance dependence are delivered separately. The forthcoming results of a randomised trial (Van Dorn et al., 2017) combining two evidence-based treatments (i.e., motivational interviewing and integrated group therapy) for substance dependence and mental health disorders aimed at increasing treatment engagement from prison to community is likely to provide insight into the feasibility of such a model for people with dual diagnosis detained in prison.

Participants who reported using crystal methamphetamine only (by any route of administration) in the week prior to their current sentence were more likely to have current poor psychiatric well-being than men who had used heroin only or crystal methamphetamine *and* heroin. The use of crystal methamphetamine has been shown to be associated with many physical and psychiatric problems, particularly psychosis (Degenhardt, & Topp, 2003; Degenhardt et al., 2008; Marshall, & Werb, 2010). In addition, for those with pre-existing mental health disorders, the impact of dependent substance use may be greater (Drake, 2007). Prison program and health services often focus resources towards people detained in prison with a complex array of health and psychosocial issues that may predispose them to recurring morbidity and reoffending. Our findings suggest that prioritising people detained in prison for mental health screening and follow-up services on the basis of types of drugs used or the types of drugs related to their offending behaviours may provide opportunities to appropriate target health service referrals in prison and in pre-release planning. This prioritisation is particularly pertinent in the context of contemporary changes in the drug use and harms among PWID. Methamphetamine has now overtaken heroin as the most commonly injected drug in Australia (Stafford, & Breen, 2017), substantial increases in the purity of methamphetamine have been detected (Scott et al., 2015) and increases have been observed in methamphetamine-related arrests and presentations of amphetamine use disorders and amphetamine psychosis at mental health services in some Australian jurisdictions (Degenhardt et al., 2017). In this context it may be beneficial to consider the development of dual service models of care that address both mental health and methamphetamine dependence simultaneously rather than have mental health and addiction specialists working with people detained in prison in relative isolation (Cumming et al., 2016). Just models may become more feasible in the future with promising methamphetamine-replacement pharmacotherapies being trialled (McKetin et al., 2017). Our findings should be considered in relation to study limitations. Self-report survey methodologies are prone to recall and social desirability biases. However, these methods among PWID have previously shown reliability relating to the collection of sensitive information (Darke, 1998; Ross et al., 1995), and have provided predictable results (Cutcher et al., 2014). The GHQ-12 has shown some variability across population groups in detecting current psychiatric symptoms (Goldberg et al., 1998; Willmott et al., 2004). However, is used widely as a screening tool for mental health referral (Hewitt et al., 2011). The findings from this study should therefore be seen in the context of identifying potential unmet need and for informing care and referral pathways, rather than the definitive identification and diagnosis of psychiatric morbidity. The authors have presented baseline (i.e, cross-sectional) data from this larger prospective cohort meaning we were unable to make causal inferences. Extensive future prospective and retrospective data linakge to health, criminogenic and social service data in this study will provide opportunities to investigate the temporal relationships between a range of exposures and mental health disorders among participants. This will also provide an opportunity to examine relationships of dual diagnosis with ongoing health, crime and reoffending risk to inform general and targeted service need.

## Conclusion

We observed a high prevalence of current poor psychiatric well-being and potential service need in a sample of incarcerated men who injected drugs regularly prior to their current sentence. Comprehensively addressing the health-related needs for this vulnerable population will require a multidisciplinary approach. Enhancing opportunities to screen and triage people detained in prison for mental health and other potential co-occurring health issues at appropriate points in their engagement with the criminal justice system will provide opportunities to better address their individual health needs and reoffending risk.
